# Patients’ Habits and the Role of Pharmacists and Telemedicine as Elements of a Modern Health Care System during the COVID-19 Pandemic

**DOI:** 10.3390/jcm10184211

**Published:** 2021-09-17

**Authors:** Patrycja Grosman-Dziewiszek, Benita Wiatrak, Izabela Jęśkowiak, Adam Szeląg

**Affiliations:** Department of Pharmacology, Faculty of Medicine, Wroclaw Medical University, Mikulicza-Radeckiego 2, 50-345 Wrocław, Poland; benita.wiatrak@umed.wroc.pl (B.W.); izabela.jeskowiak@umed.wroc.pl (I.J.); adam.szelag@umed.wroc.pl (A.S.)

**Keywords:** COVID-19 pandemic, telemedicine, e-health, pharmacist, vaccination, pharmaceutical care

## Abstract

Aims/Introduction: The Polish government introduced the epidemic on 20 March 2020, after The World Health Organization (WHO) announced the new coronavirus disease (COVID-19) in January 2020. Patients’ access to specialist clinics and family medicine clinics was limited. In this situation, pharmacists were likely the first option for patient’s health information. On 18 March 2020, the National Health Fund issued modifications that increased the accessibility to primary health care such as telemedicine. The development of e-health in Poland during the COVID-19 pandemic included the implementation of electronic medical records (EDM), telemedicine development, e-prescription, and e-referrals implementation. We investigated this emergency’s effect on patients’ health habits, access to healthcare, and attitude to vaccination. Materials and methods: An anonymous study in the form of an electronic and paper questionnaire was conducted in March 2021 among 926 pharmacies patients in Poland. The content of the questionnaire included access to medical care, performing preventive examinations, implementation of e-prescriptions, patient satisfaction with telepathing, pharmaceutical care, and COVID-19 vaccination. Results: During the COVID-19 pandemic, 456 (49.2%) patients experienced worse access to a doctor. On the other hand, 483 (52.2%) patients did not perform preventive examinations during the COVID-19 pandemic. Almost half of the patients (45.4% (*n* = 420)) were not satisfied with the teleconsultation visit to the doctor. A total of 90% (*n* = 833) of the respondents do not need help in making an appointment with a doctor and buying medications prescribed by a doctor in the form of an e-prescription. In the absence of access to medical consultation, 38.2% (*n* = 354) of respondents choose the Internet as a source of medical advice. However, in the absence of contact with a doctor, 229 persons (24.7%) who took part in the survey consulted a pharmacist. In addition, 239 persons (25.8%) used pharmacist advice more often during the COVID-19 pandemic than before its outbreak on 12 March 2020. Moreover, 457 (49.4%) respondents are satisfied with the advice provided by pharmacists, and even 439 patients of pharmacies (47.4%) expect an increase in the scope of pharmaceutical care in the future, including medical advice provided by pharmacists. Most of the respondents, 45.6% (*n* = 422), want to be vaccinated in a hospital or clinic, but at the same time, for a slightly smaller number of people, 44.6% (*n* = 413), it has no meaning where they are will be vaccinated against COVID-19. Conclusions: Telemedicine is appreciated by patients but also has some limitations. The COVID-19 pandemic is the chance for telemedicine to transform from implementations to a routine healthcare system structure. However, some patients still need face-to-face contact with the doctor or pharmacist. Pharmacists are essential contributors to public health and play an essential role during the COVID-19 pandemic. Integration of pharmaceutical care with public health care and strong growth in the professional group of pharmacists may have optimized patient care.

## 1. Introduction

The World Health Organization (WHO) announced the new coronavirus disease (COVID-19) in January 2020, which was declared a pandemic on 20 March 2020 [1]. The Polish government introduced the epidemic on 20 March 2020 [2]. The coronavirus epidemic made it necessary to introduce solutions that would enable the patient to obtain medical services at a distance, thus reducing the risk of infection through contact with patients, e.g., in the waiting rooms of the clinic. To prevent infection, regulations such as physical distance, wearing a mask, and hand disinfection were introduced. It was also recommended that the rooms be well ventilated and to avoid crowds.

From 12 March 2020, educational institutions, universities, cinemas, theaters, and other cultural centers in Poland were closed. These were preventive measures related to the spread of the coronavirus. Moreover, patients’ access to specialist clinics and family medicine clinics was limited. Various kinds of telemedicine have developed in Poland for many years, including telerehabilitation [3], telegeriatrics [4], and telepathology [5], but the pandemic resulted in faster development of services in this area. Medical specialists, apart from health care institutions, provided teleconsultation. Patients of oncology, cardiology, and neurology clinics that are already under the constant care of specialists and require periodic checks, also go to telemedicine. Telemedicine has reduced the risk of the disease spreading and provided greater safety for both patients and medical staff. On 18 March 2020, the president of the National Health Fund issued an ordinance amending the ordinance on the conditions for concluding and implementing contracts for healthcare services in the field of primary healthcare. The introduced modifications increased the accessibility to primary health care. The patients were informed about the possibility of obtaining teleconsultation without the need for a personal visit.

One of the main assumptions of the European Union is the development of the economy based on modern information technologies. This also applies to health care, where modern solutions increase the effectiveness of health care. For example, the development of e-health in Poland includes information and telecommunications technologies to support activities related to health protection, implementation of electronic medical records (EDM), e-prescription implementation, implementation of e-referrals, and development of telemedicine [6].

According to the International Pharmaceutical Federation (FIP), the global leadership body for pharmacists, pharmaceutical scientists, and pharmaceutical educators, coordinated and produced as of January 2020 an international response to the COVID-19 pandemic and can advise and support, including therapeutic options and vaccines, diagnostic testing, infection prevention, and control, supply chain of medicines and medical products and education [7].

The role of health institutions, including pharmacies, is to maximize the protection of the elderly during the COVID-19 pandemic. This is the special social group constantly stocking up on medicines due to the multi-disease burden characteristic of this group. Therefore, enabling family members of the elderly, their friends, neighbors, or even volunteers to fulfill prescriptions for these people is a manifestation of responsibility, an expression of care for the health and life of seniors by limiting their exposure to pathogens in a pharmacy or pharmacy point, or on their way to them.

The next change in the healthcare system during the COVID-19 pandemic in Poland was the regulation of the Minister of Health of 15 February 2021, on the qualification of persons carrying out preventive vaccinations against COVID-19, physiotherapists, pharmacists, and laboratory diagnosticians can carry out vaccination protective against COVID-19. As a result, pharmacists, physiotherapists, and laboratory diagnosticians, after specialist training, constituted new human resources authorized to carry out vaccination against COVID-19 and supported vaccination points in vaccination against COVID-19. The regulation of the Minister of Health of 15 February 2021 has also identified group zero of the National Immunization Program to be vaccinated against COVID-19 first. The study of post-vaccination reactions and vaccination propensity in the group of the healthcare sector (doctors, dentists, pharmacists) was conducted in the Polish population. The results illustrate the enormous interest in vaccination in the health sector [8].

The study aimed to evaluate how the COVID-19 pandemic influences patients’ health habits, access to healthcare, and attitude to vaccination.

## 2. Materials and Methods

### 2.1. Study Design

An anonymous study in the form of an electronic and paper questionnaire was conducted in March 2021 among 926 pharmacy patients in Poland. Survey-based on Google^®^ Forms to collect data. The participation was voluntary and anonymous. The paper version of the survey was intended only for people who do not use electronic devices, and its use was very limited due to sanitary reasons during the COVID-19 epidemic. The questionnaire can be found as Supplementary Materials Attachment S1. The questionnaire was conducted by the pharmacist with social distancing, and then the results were entered into an electronic questionnaire.

### 2.2. Ethical Approval

The study has been performed following the ethical standards laid down in the 1964 Declaration of Helsinki and its later amendments. The study was approved by the Bioethics Committee of the Wroclaw Medical University (KB-253/2021).

### 2.3. Description of the Questionnaire

The questionnaire consisted of 16 questions. The first section of the survey explored demographic and education characteristics. The demographic variables included sex (male, female), age (divided into groups 19–30, 31–40, 41–50, 51–60, 61–70, 71–80, and over 80 years of age), education (higher, secondary, primary, vocational, student). The content of the questionnaire also included medications taken for chronic disease, access to medical care and performing preventive examinations during the COVID-19 pandemic, implementation of e-prescriptions, patient satisfaction with telepathing, and sources of medical knowledge for patients in case of lack of contact with a doctor (Internet, pharmacist, nurse, friends, family, physiotherapist).

We asked about patients’ participation in pharmaceutical care consultations and willingness to extend the scope of pharmaceutical care, satisfaction with advice provided by the pharmacist, and place of COVID-19 vaccination (hospital or ambulatory, pharmacy, not relevant, I do not want to be vaccinated).

### 2.4. Statistical Analysis

Statistical analyzes were performed with Statistica v13.0. Pearson’s chi-square test was used to compare the differences between the different subgroups.

## 3. Results and Discussion

### 3.1. Population Characteristics

**Figure 1:**
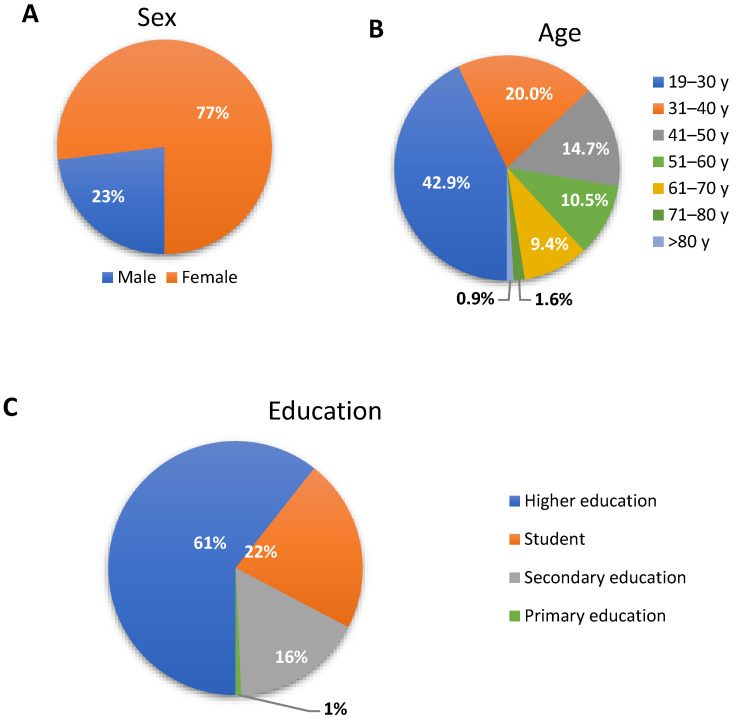
The characteristics of the study group. (**A**) Sex, (**B**) Age (y—years), (**C**) Education.

We performed a study of patients aged 18 years and older. Our total data included 926 patients opinion, 76.9% (*n* = 712) of participants were females, 23.1% (*n* = 214) were males. More women took part in the survey, but the groups of women and men were considered separately in the statistical analysis. When asked about their education, the majority of the survey participants had higher education (*n* = 552, 59.6%), while 21.7% (*n* = 201) were students, 16.3 (*n* = 151) had secondary education, 6 (0.6%) had primary and 1.7% (*n* = 16) had vocational. A total of 43% (*n* = 398) of people who took part in the survey are aged 19–30, 20% (*n* = 185) are people in the 31–40 age group, 14.7% (*n* = 136) in the 41–50 age group, 10.5% (*n* = 97) in the 51–60 age group, 9.4% (*n* = 87) in the 61–70 age group, 1.6% (*n* = 15) in the 71–80 age group and only 0.9% (*n* = 8) over 80 years of age (Figure 1). The elderly patients are mostly not Internet users and need a paper version of the survey, which was high-risk during the COVID-19 pandemic.

### 3.2. Patients Health

**Figure 2:**
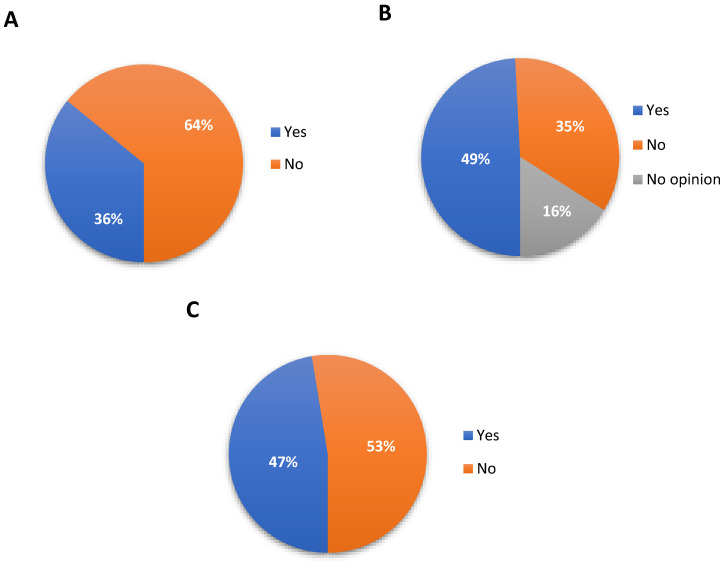
Health habits of patients. (**A**) Taking drugs in chronic disease, (**B**) Worsening access to a doctor during a pandemic, (**C**) Performing preventive laboratory diagnostics.

The largest number, 64.1% (*n* = 594) of respondents not taking medications for chronic disease, while 35.9% (*n* = 332) are constantly taking medication (Figure 2). Statistically, in the studied population, women suffer from chronic diseases more often. This relationship was calculated separately for the group of women and men. It was evaluated that women reported higher differential rates of anxiety and depression than men [9]. Moreover, women showed greater mean heart rate and showed greater vagal activity: heart rate power of heart rate variability [10]. A total of 49.2% (*n* = 456) of patients notice that they have worse access to a doctor during the COVID-19 pandemic. However, for 34.8% (*n* = 322), the access to a doctor has not worsened. Women suffer from worsening access to a doctor more often than men. A total of 52.2% (*n* = 483) of patients not performing preventive examinations during the COVID-19 pandemic, while 47.1% (*n* = 436) carries out preventive examinations even during pandemic.

The COVID-19 pandemic has affected the global population, including patients with chronic diseases such as hypertension, diabetes, arthritis, asthma, cancer [11]. A high incidence of chronic diseases and a high level of disability among older adults in Poland were observed [12]. Patients with chronic diseases require constant contact with a doctor, taking medications, and regular examinations. It was reported that patients with diabetes during the COVID-19 pandemic presented behavioral changes and needed adequate support to maintain appropriate glycemic control [13]. In a group of patients with small fiber neuropathy, pandemic distress impacted physical health. Moreover, the higher levels of disability were associated with suffering from changes in the neurologist-patient relationship. Patients with migraines during the COVID-19 pandemic complained of agitation and anxiety [14]. Patients with chronic diseases suffered from changes in medical care during the COVID-19 pandemic and need frequent access to health facilities, including in-person visits, follow-ups, and treatments [15].

### 3.3. Telehealth

Almost half of the patients were not satisfied with a doctor’s appointment in teleconsultation (45.4% (*n* = 420)). However, it was the most frequently chosen response by patients. A total of 29.4% (*n* = 272) of patients had no opinion, and 25.3% (*n* = 234) were satisfied with medical consultation in the form of teleconsultation. Our research shows that women were more often satisfied. We have shown a correlation between patient satisfaction with teleconsultation in a group of people who are constantly taking medication (*p* < 0.01, *r* = 0.9). The demonstration of this relationship is probably related to the need in the group of patients with chronic diseases only to ask for a prescription for constantly used drugs. Interestingly, it was observed that dissatisfaction with teleconsultation was most often declared by patients in the 19–30 age group. This is the opposite of research from Norway. However, it should be noted that the study did not recommend the use of e-consultation in assessing acute exacerbations and the emergence of new health problems that require re-examination [16]. This may be because younger people are less likely to have chronic diseases. Studies carried out by another research group have shown that people suffering from chronic diseases accompanied by pain do not feel satisfied with the solutions offered by telemedicine [17].

**Figure 3:**
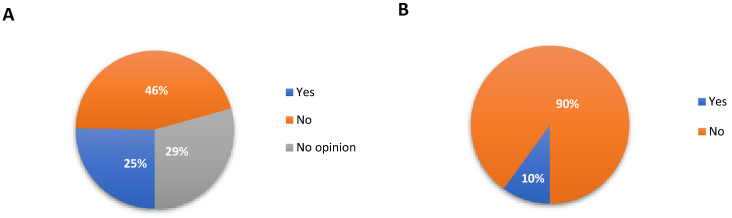
Satisfaction with a doctor’s appointment in the form of teleconsultation, e-prescription, and need for assistance in purchasing prescription drugs from e-prescription in pharmacy and making an appointment with a doctor. (**A**) Satisfaction with teleconsultation, (**B**) The need for assistance when using teleconsultation or e-prescription.

Most of patients (86.2% (*n* = 798)) are satisfied with the e-prescriptions introduction. The greatest satisfaction with the form of e-prescriptions (86%) is observed in the 19–30 and 31–40 age groups (Figure 3). In the 41–50 age group, it is also high; 80% of respondents are satisfied with e-prescriptions, but over 50 years of age, satisfaction with e-prescriptions decreases. This may prove that older people find it more difficult to use electronic tools.

For the largest number of 90% (*n* = 833) respondents, there was no need to help with prescription drugs from e-prescription in pharmacy and making an appointment with a doctor. It is surprising that only until the age of 30 do patients not declare the need for help. Over 40, had been an increasing need to help with the technology of telemedicine. It depends not only on age but also on the level of education. Previous research has shown that users of digital health services in primary care are more likely to be younger adults, women, and digitally active users with higher education [16].

Telehealth uses electronic information and telecommunication technologies to support long-distance clinical health care, patient and professional health-related education, public health, and health administration [18,19]. Telemedicine, including telephone consultation, short message services, and video conferencing, is used worldwide during the COVID-19 pandemic. Health care systems implemented telemedical solutions used to care for patients with COVID-19 staying at home, with new solutions minimizing the time of direct patient contact with the doctor and the infection risk [20]. The benefit of telemedicine services for surgical patients was proved [21]. According to the National Health Fund and the Ministry of Health report, most of the doctors’ visits were telephone consultation (81.5%), while only 0.3% was video consultation. A total of 97.8% of the respondents declared that the doctor properly and satisfactorily took care of the patient by handing over the necessary documents (prescriptions, referrals, and recommendations). After teleconsultation, about 80% of patients make an appointment with a primary care physician, and 92% of patients purchase drugs from e-prescription issued during teleconsultation [22].

### 3.4. Access to Health Information and Pharmacist Advice

**Figure 4:**
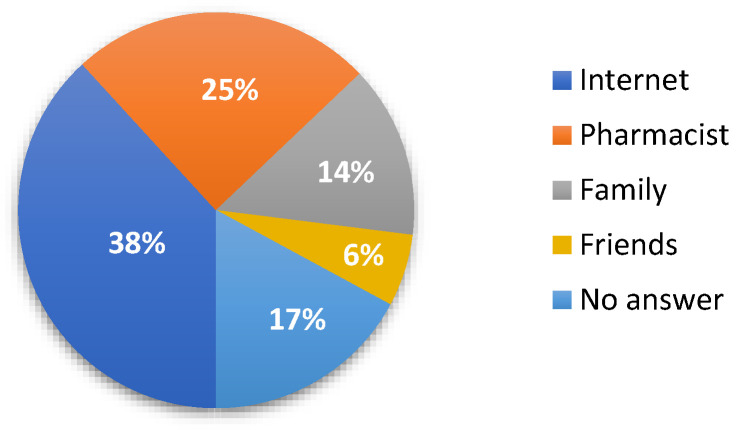
Source of medical advice in case of lack of contact with a doctor.

Of 926 participants who completed the questionnaires, 38.2% (*n* = 354) declared the Internet a source of medical advice without contact with a doctor. For 24.7% (*n* = 229) patients, the source of medical advice is the pharmacist. A total of 14.1% (*n* = 131), and 5.9% (*n* = 55) declared family and friends (Figure 4).

A total of 25.8% (*n* = 239) of patients more frequently used pharmacist advice during the COVID-19 pandemic. This may be due to limited access to a doctor.

A significantly higher percentage of patients is satisfied with the advice provided by a pharmacist (49.4% *n* = 457 vs. 6.7% *n* = 62, *p* < 0.05). Moreover, 47.4% (*n* = 439) of patients expect an increase in the advice provided by a pharmacist in the future.

As healthcare professionals with high public availability, it was reported that community pharmacists are likely the first option for patient’s health information [23]. During COVID-19, lockdown pharmacies were the essential services that should keep working [24]. The pharmacist’s role is education on infection control and preventive measures to reduce virus transmission (e.g., hand hygiene, social distancing, self-isolation) during COVID-19 [25]. Implemented social distancing and advice people with health issues to stay at home may cause a lack of contact between patients and healthcare providers. The pharmacy pharmacists provided patients information and education materials that may support communication, especially for vulnerable patients [26]. Moreover, pharmacist pharmacists should pay particular attention to the emotional situation of patients [27]. In another survey, 399 from 622 Spain patients strongly agreed with the sentence “I felt comfortable going to a community pharmacy to receive testing for COVID-19 infection”. Many patients value the friendliness, professionalism, and knowledge of the pharmacists [28]. It was clearly demonstrated that the pharmacist interventions were reducing readmissions and supporting patient safety [29,30].

During the COVID-19 pandemic, pharmacists in Poland were given the possibility of issuing pharmaceutical prescriptions for themselves and some family members, “pro familiae prescription” as e-prescriptions and the possibility of writing a pharmaceutical prescription for patients in the event of health endangerment. These prescriptions are 100% payment, with limitations up to 180 days of therapy [31]. The next step to optimize patient care during the COVID-19 pandemic included pharmacists accessing core clinical and medication information (the patient’s Internet account IKP) with the patient’s consent. The purpose of access the patient’s Internet account IKP for pharmacists or pharmacies can improve possible adverse drug interactions detection, identify a cheaper alternative to a medicinal product, or may also be the basis for issuing a pharmaceutical prescription [32].

The Ministry of Health points to the drug review service as the first pharmaceutical care service to be introduced in Polish pharmacies. The pilot in this area will be directed to patients burdened with multidrug use. They will aim to detect drug problems and improve the clinical effectiveness of pharmacotherapy [33]. In addition, increased emphasis on educational campaigns among pharmacists and their patients may be needed. Pharmacies are part of the primary care system. In addition, there has been skepticism about pharmacist collaboration with other healthcare professionals due to their isolated roles and the commercial aspect [34]. Nevertheless, pharmacists can play a significant role in recommending symptom management for mild conditions, making sure medications are refilled on schedule, and reduce unnecessary hospital visits, where individuals might be exposed to COVID-19 [35]. As the global leadership body for pharmacists, the International Pharmaceutical Federation (FIP) responds to the COVID-19 pandemic identified therapeutic options and vaccines, diagnostic testing, and infection prevention and control as main areas of work [7].

### 3.5. COVID-19 Vaccine

Most of the patients, 45.6% (*n* = 422), wanted to be vaccinated in a hospital or ambulatory, but for almost the same group, 44.6% (*n* = 413) place of COVID-19 vaccination is not relevant. However, it was no statistical significance. A total of 7.1% (*n* = 66) of the patients did not want to be vaccinated.

In the USA, pharmacists have been authorized to administer vaccinations since 2009 [36]. Most pharmacists are trained to administer a majority of FDA-approved vaccinations to all patients. Moreover, pharmacists improved influenza vaccination rates [37]. In Europe, vaccinations are administered in pharmacies in 13 countries, for example, in Great Britain, Portugal, and Norway [38]. In Poland, from 15 June 2021, the National Health Fund began recruiting pharmacies that want to participate in the National Vaccination Program. Under the program, licensed pharmacists working in qualified pharmacies will vaccinate patients against COVID-19. In order to be able to vaccinate against COVID-19, pharmacists must complete a two-stage course consisting of a theoretical and practical part. According to the Polish Pharmaceutical Chamber, almost nine thousand. Pharmacists have already acquired the authorization to vaccinate against COVID-19, and approximately 6.3 thousand pharmacists can make qualifications in Poland [39]. To achieve herd immunity, more than 60% of the population needs to be vaccinated. This is a significant challenge for current healthcare systems [40]. Increasing the number of vaccination points by including pharmacies in the program will improve the rate of vaccinated people. The Chief Sanitary Inspector in Poland (source: www.gov.pl/web/szczepimysie (accessed on 22 June 2021)) reported the administration of 26,665,528 doses of vaccines and 11,337,728 fully vaccinated people. However, in a situation where many people remain unvaccinated, telemedicine is the safest solution in primary care.

This study has some limitations. The study population was young, limited to Internet-using participants. It was necessary for safety reasons during the COVID-19 pandemic. Larger studies with older patients who especially are not Internet users have to be conducted. COVID-19 pandemic also changed patient priorities. During the pandemic, patients may be less likely to see a doctor for chronic conditions, which could affect patient satisfaction [41].

## 4. Conclusions

Telemedicine is appreciated by patients but also has some limitations. The COVID-19 pandemic is creating a challenge for doctors, patients, and pharmacists. Telemedicine has the chance to transform from implementations to a routine healthcare system structure. However, some patients still need face-to-face contact with the doctor or pharmacist. Pharmacists are essential contributors to public health and played a key role during the COVID-19 pandemic. Integration of pharmaceutical care with public health care and a strong increase in the professional group of pharmacists may have optimized patient care and impact the budget.

It should also be noted that the development of e-health must not be at the expense of discriminating against older people, digitally excluded, deaf, or with other disabilities. The role of doctors and pharmacists is to care for these particular groups of patients properly.

## Data Availability

The data presented in this study are available within the article.

## Supplementary Materials

The following are available online at https://www.mdpi.com/article/10.3390/jcm10184211/s1, Attachment S1: survey questionnaire.
