# Supplementary material for: Patients’ Habits and the Role of Pharmacists and Telemedicine as Elements of a Modern Health Care System during the COVID-19 Pandemic
Source: J Clin Med. 2021 Sep 17;10(18):4211. doi: 10.3390/jcm10184211 (PMC8470917; doi:10.3390/jcm10184211)
Supplement: Supplementary file 1 [file jcm-10-04211-s001.zip › jcm-1297263-SI.pdf]

Patient assessment of access to healthcare during a COVID 19 pandemic

Please complete an anonymous questionnaire consisting of 20 short questions regarding, among others, getting COVID 19, post-vaccination reactions after receiving the first and second doses of the COVID19 vaccine, and motivation to proceed with vaccination. The survey is conducted by the team of the Chair and Department of Pharmacology at the Medical University of Wrocław.

1. Sex:
☐woman
☐man
2. Age
☐ 19-30 years
☐ 31-40 years
☐ 41-50 years
☐ 51-60 years
☐ above 60 years
3. Education:
☐Higher education
☐Secondary education
☐Primary education
☐Vocational education
☐Student
4. Are you constantly taking medication because of a chronic disease?
☐Yes
☐No
5. Did your access to a doctor worsen during the COVID 19 pandemic?
☐Yes
☐No
☐No sure
6. Do you perform performing preventive examinations during the COVID-19 pandemic?
☐Yes
☐No
☐No sure
7. Do e-prescriptions make it easier for you to fulfill your prescriptions?
☐Yes
☐No
☐No sure
8. Are you satisfied with the doctor's appointment in the form of teleconsultation?
☐Yes
☐No
☐No sure
9. Do you need help to arrange a medical appointment in the form of teleconsultation and to receive or complete an e-prescription?
☐Yes
☐No
☐No sure
10. In the absence of contact with a doctor, the source of medical advice for you is:
☐ Internet

☐ Pharmacist
☐ Nurse
☐ Friend
☐ Family
☐ Physiotherapist
☐ Other
11. Do you take more dietary supplements during the pandemic than before the COVID 19 epidemic?
☐Yes
☐No
☐No sure
12. What dietary supplements do you take?
☐ I don’t take any supplements
☐ Supplements with vitamin C
☐ Supplements with vitamin D
☐ Multivitamin supplements
☐ Supplements with magnesium
☐ Supplements with zinc
☐ Supplements with Omega 3 fatty AIDS
☐Other
13. During a pandemic, do you use the pharmacist's advice in a pharmacy more often?
☐Yes
☐No
☐No sure
14. Are you satisfied with the advice provided by pharmacists?
☐Yes
☐No
☐No sure
15. Do you expect an increase in the scope of advice provided by pharmacists in the future?
☐Yes
☐No
☐No sure
16. Where would you like to be vaccinated against COVID19?
☐ Hospital or ambulatory
☐ Pharmacy
☐Not relevant
☐ I do not want to be vaccinated
